# Risk Beyond the Variant: A Composite Metric for Autism Gene Pathogenicity

**DOI:** 10.3390/genes17080892

**Published:** 2026-07-29

**Authors:** Beloslava Malakova, Simeon Karpuzov, Dobromir Tsolyov, Georgi Petkov, Milen Zamfirov

**Affiliations:** 1 GATE Institute, Sofia University, 1164 Sofia, Bulgaria; beloslava.malakova@gate-ai.eu (B.M.); simeon.karpuzov@gate-ai.eu (S.K.); dobromir.tsolyov@gate-ai.eu (D.T.); 2 Faculty of Educational Sciences and Arts (FNOI), Sofia University, 1574 Sofia, Bulgaria; m.zamfirov@fppse.uni-sofia.bg

**Keywords:** autism spectrum disorder, genes, genome, pathogenicity, risk score, revel, Hardy–Weinberg heterozygosity

## Abstract

**Background**: Variant-based pathogenicity predictors such as REVEL evaluate missense variants in isolation, discarding the gene-length and allele-frequency context needed to compare collections of genes. **Methods**: We introduce a composite gene-level metric integrating Hardy–Weinberg heterozygosity, coding-sequence length, and REVEL scores. The metric returns a single value per gene expressing variant burden per unit of coding sequence within a given cohort, so that the ratio between a case and a control cohort quantifies gene-level enrichment. It was evaluated on 55 high-confidence autism genes, defined as the intersection of three large-scale ASD sequencing studies, against the 1000 Genomes reference. **Results**: It identifies elevated pathogenic burden in 48 of 55 genes, removes gene-length and variant-count confounds, and substantially outperforms naive gene-level aggregation of REVEL scores. Bootstrap resampling and a label-permutation control confirm the enrichment is stable and not an artefact of the scoring construction. **Conclusions**: The metric allows genes to be ranked within a set and aggregate burden to be compared across gene sets. We present it as a complementary gene-level layer for case–control and gene-set comparisons, with a nonlinear successor outlined as future work.

## 1. Introduction

### 1.1. Motivation

The genetic architecture of autism spectrum disorder [1,2,3] (ASD) is among the most complex in human medicine. Large-scale sequencing studies have identified hundreds of rare variants distributed across thousands of genes, but the field still lacks a principled method for determining how pathogenically loaded a specific gene set is in affected individuals compared to the general population. Answering this requires moving from the variant level, where most current tools operate, to the gene level and finally the gene-set level. The present work introduces a composite metric designed to make that transition. This topic matters because the underlying scientific questions are framed at the level of gene sets and their evolutionary history rather than at the level of individual variants. ASD-associated genes are subject to strong purifying selection, since de novo variants in highly constrained genes are a major source of severe phenotypes, and yet ASD persists in the population at stable proportions. De novo events are not, however, the only contributor. Maternally inherited variants in X-linked genes contribute to the male preponderance of ASD, inherited variants frequently show incomplete penetrance in carrier parents, and the cumulative burden of inherited rare variants adds substantially to overall risk. This inherited component is itself a gene-level phenomenon, since individually low-penetrance variants become interpretable only in aggregate. Whether the pathogenic burden carried by a gene increases steadily from ancestral populations through the contemporary general population to disease cohorts, or whether it fluctuates, bears directly on whether a gene is under sustained purifying selection or balancing selection. Questions such as these cannot be answered with a score that evaluates one variant at a time. They require a quantity that can be computed consistently across whole genes and compared across cohorts of different sizes and sequencing strategies.

The dominant paradigm in computational variant interpretation is per-variant pathogenicity prediction. Tools such as SIFT [4], PolyPhen-2 [5], MutationTaster [6], and CADD (Combined Annotation-Dependent Depletion) [7] assign a deleteriousness score to each individual missense substitution based on evolutionary conservation, physicochemical properties of the amino acid change, and structural context. REVEL (Rare Exome Variant Ensemble Learner) [8] extends this approach by combining 13 individual tools into an ensemble score calibrated specifically for rare missense variants, achieving State-of-the-Art performance on ClinVar [9] benchmarks. REVEL scores range from 0 to 1, with higher values indicating greater predicted pathogenicity, and have become a standard annotation layer in rare disease genomics pipelines. These tools are valuable, but they are instruments rather than answers to a gene-level scientific question: each evaluates a variant in isolation. A given REVEL score carries the same meaning regardless of whether the variant lies in a short or a long gene, whether the allele is exceedingly rare or relatively common, and whether the gene is absolutely intolerant of loss-of-function or freely tolerates it. This is appropriate for classifying a single mutation in a single patient, but can become a limitation the moment a comparison of pathogenic burden is carried out across genes or between cohorts. The natural way forward is therefore not to discard REVEL but to use it as a component within a measure that also captures the context around each variant.

### 1.2. Related Works

Two factors are particularly damaging for cross-gene comparison. The first one is gene length. Longer genes present more target sequence to mutation and therefore accumulate more rare variants by chance in any sequenced cohort. A basic summation of REVEL scores across variants in a gene will be inflated for long genes simply because they contain more variants, not because their variants are more damaging. The second factor is allele frequency. A variant observed in a small portion of the general population contributes far less to disease burden than a variant observed in a single proband, yet both receive a REVEL score based solely on their predicted amino acid effect. Without frequency weighting, common benign variants and rare pathogenic ones are treated equivalently in any gene-level aggregate. Genes that are both long and tolerant of variation will accumulate many common, low-pathogenicity variants in population reference databases, generating high REVEL sum scores. This has the practical consequence of making established disease genes with compact coding sequences appear less pathogenic than large, biologically peripheral genes—any gene-level or gene-set metric that does not explicitly correct for length and frequency, and that measures sequencing depth and gene size as much as it measures pathogenicity.

A further blind spot common to per-variant tools is the absence of gene-network context: a moderately damaging variant in a central regulatory gene may be more consequential than a high-scoring variant in a peripheral gene, yet variant-level scores cannot express this.

Several metrics exist at the gene level, though none directly addresses the cross-cohort comparison problem. The pLI score [10] (probability of loss-of-function intolerance) and its successor LOEUF (loss-of-function observed) from gnomAD (Genome Aggregation Database) [11] quantify a gene’s evolutionary intolerance to protein-truncating variants. These are powerful priors about gene function but are population-derived constants, not scores that can be computed on a new case cohort and compared to a control. Similarly, haploinsufficiency scores [12] and the missense tolerance ratio (MTR) [13] characterize gene-level constraint but do not produce a quantity that is directly comparable between a disease cohort and a reference panel. Gene-set enrichment approaches such as GSEA (Gene Set Enrichment Analysis) [14] and its genomic adaptations test whether a defined gene set is over-represented among top-ranking variants, but the ranking itself is typically a per-variant score, inheriting all the confounds described above. Burden tests such as SKAT (Sequence Kernel Association Test) [15] or CMC (Combined Multivariate and Collapsing) [16] aggregate variant information across a gene and test for association with a phenotype, but they require individual-level genotype data and a sufficiently large sample size to achieve statistical power. They are not designed to produce an interpretable measure of gene pathogenicity that can be compared across studies or cohorts of different sizes.

ASD provides an ideal test case for a gene-level metric because its genetic architecture spans the full spectrum of variant effects. SFARI (Simons Foundation Autism Research Initiative) Gene [17] curates high-confidence ASD-associated genes into evidence categories. High-confidence ASD genes such as SCN2A, CHD2, SYNGAP1, and KMT2C are supported by multiple independent de novo variant studies. The 1000 Genomes Project [18] provides a well-characterized general-population reference panel in which the same genes can be queried for rare missense variant burden. The contrast between these two cohorts offers a well-characterized positive control for evaluating whether a gene-level metric correctly identifies elevated pathogenic burden in known disease genes. The evolutionary context adds further motivation. ASD-associated genes are subject to strong purifying selection, as de novo variants in high-pLI genes are a major source of severe phenotypes. ASD, however, persists in the population at stable prevalence. Understanding whether the pathogenic variant burden in a gene increases monotonically from ancient populations through the general population to disease cohorts, or whether it fluctuates, has implications for distinguishing genes under purifying selection from those under balancing selection. This requires a metric that can be applied consistently across cohorts with different sizes, sequencing strategies, and time depths.

### 1.3. Paper Contributions and Organization

We present a composite gene-level pathogenicity metric that integrates Hardy–Weinberg expected heterozygosity [19], coding sequence length, and per-variant REVEL scores into a single additive quantity. The Hardy–Weinberg term weights each variant by the expected frequency of heterozygous carriers, encoding the principle that rare variants contribute disproportionately to disease burden; division by gene length removes the size confound; and multiplication by REVEL acts as a pathogenicity filter that separates rare benign from rare damaging variants. Concretely, the contributions of this work are as follows:

- A composite metric that, by construction, removes the gene-length and variant-count confounds that make per-variant scores unsuitable for cross-gene comparison, and that is computable on any cohort with variant calls and allele frequencies.
- A decomposition of the metric into its allele-frequency-and-length and REVEL components, evaluated on 55 high-confidence ASD genes derived from three large-scale studies against a 1000 Genomes control, which isolates the contribution of each component and shows transparently where the REVEL term does and does not add discriminative power.
- Empirical evidence that the composite metric identifies elevated pathogenic burden in 48 of 55 known ASD genes and provides a substantially wider dynamic range than REVEL-based scoring alone, making it suitable for ranking genes within a set.
- An internal validity framework combining bootstrap confidence intervals and a label-permutation negative control that requires no external gene set, establishing that the observed enrichment is stable and is not an artifact of the scoring construction.

### 1.4. Paper Organization

The next section sets out the methodology, which includes the following subsections:

- Derivation of the formula;
- The three scoring schemes;
- The datasets;
- The statistical framework.

The third section describes data preparation and presents the results, covering the removal of structural confounds, cohort-level discrimination, dynamic range, and robustness analyses. The final section summarizes the findings, situates the proposed metric relative to existing approaches, and discusses its limitations, together with a concrete roadmap for a nonlinear, evolutionarily informed successor metric applied to whole-genome data.

## 2. Materials and Methods

### 2.1. Overview

The methodological contribution of this work is a gene-level composite pathogenicity metric that aggregates information from rare missense variants across all variants in a gene into a single, comparable score. The metric is designed to satisfy three requirements that existing per-variant tools do not simultaneously meet: it must be computable on any cohort for which variant calls and allele frequencies are available, it must be directly comparable across genes of different lengths and variant densities, and it must separate the pathogenicity signal of rare damaging variants from the noise introduced by rare benign variants and common polymorphisms. This section describes the derivation of the formula, the three scoring schemes compared, the datasets used, and the statistical framework for evaluating formula performance.

### 2.2. Selection of Relevant Genes

**Figure 1:**
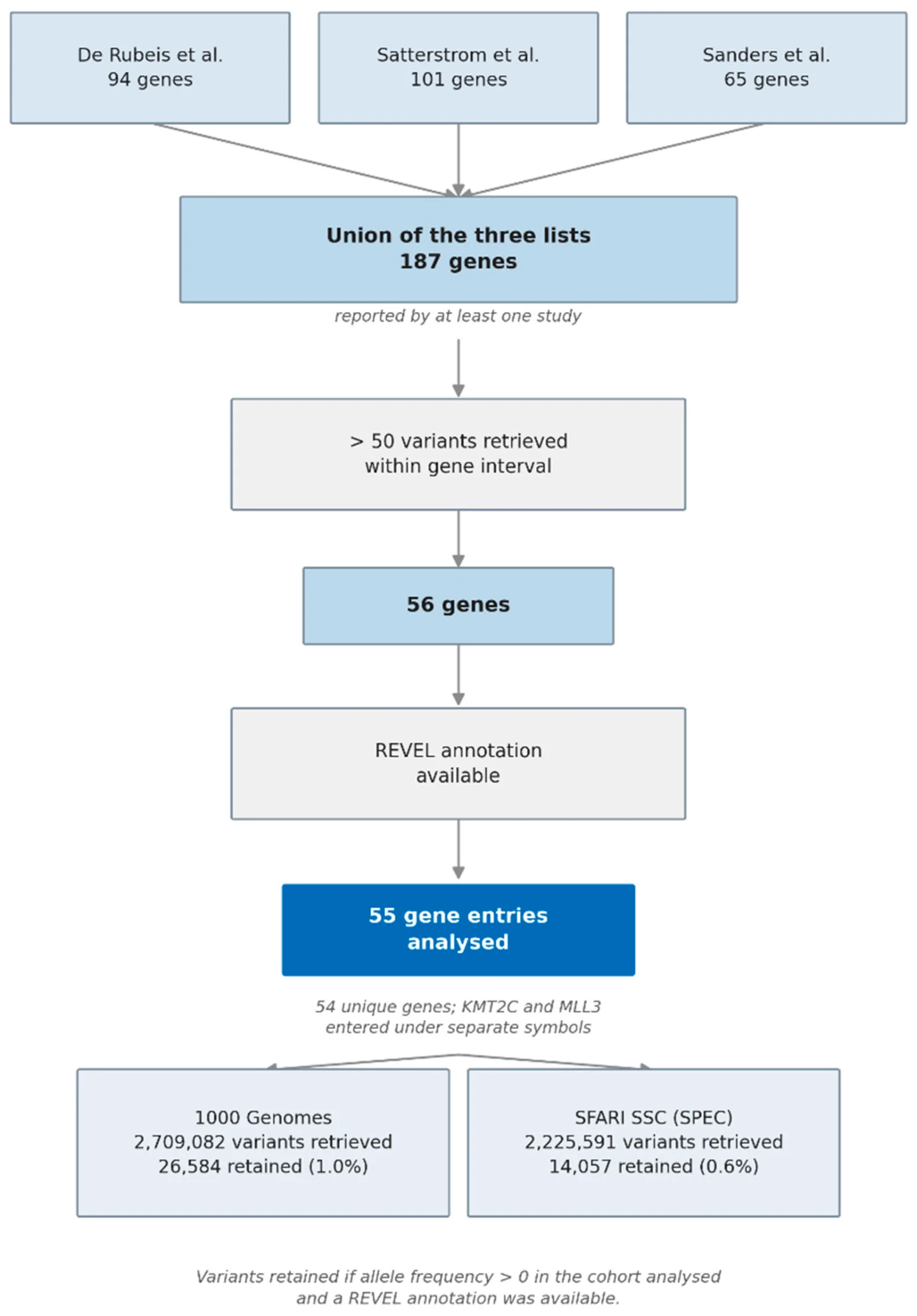
Gene-selection scheme. Genes reported as ASD-associated were extracted from three large-scale sequencing studies and combined, yielding 187 genes in total. Two successive filters were applied: genes were required to carry more than 50 variants retrieved within the gene interval (56 genes), and to have variants with available REVEL annotations (55 entries). The lower panels give the number of variants retrieved and retained within the selected gene intervals for each cohort; the low retention fraction reflects that REVEL is defined only for missense substitutions [20,21,22].

The gene set was assembled from three large-scale ASD sequencing studies selected for being highly cited, methodologically independent landmark analyses, each explicitly reporting a list of ASD-associated risk genes: Sanders et al. [20] (65 genes), Satterstrom et al. [21] (101 genes), and De Rubeis et al. [22] (94 genes). Gene symbols were extracted from each publication and combined as a union, yielding 187 genes reported as ASD-associated by at least one study. Two successive filters were then applied. First, genes were required to carry more than 50 variants within the datasets analyzed here, ensuring sufficient variant coverage for stable per-gene estimation, which retained 56 genes. Second, genes were required to have variants carrying REVEL annotations, since REVEL is a required input to the composite metric; this removed one further gene and produced the final panel of 55 entries. The complete panel is listed in Appendix A as Appendix List A1, and the three source-gene lists, together with their pairwise and three-way intersections, are provided as Appendix List A2. We note that this procedure prioritizes variant coverage over cross-study consensus. Because the union rather than the intersection of the three studies was used, and because the variant-count threshold subsequently removed genes sparsely represented in these datasets, several widely reported ASD genes are not included in the final panel. The panel should therefore be understood as a set of ASD-associated genes with sufficient variant coverage for this analysis, rather than as a consensus high-confidence gene set. The procedure is graphically illustrated in Figure 1.

### 2.3. Datasets

Two variant datasets were assembled for the 55 selected genes. The first is the SFARI SSC whole-genome sequencing cohort [23] (referred to throughout as SPEC), comprising rare variants identified in ASD probands from the Simons Simplex Collection and obtained through SFARI Base. For each gene, variants were filtered to missense consequences, annotated with REVEL scores where available, and summarized at the gene level as a total variant count and a sum of REVEL scores.

The second is the 1000 Genomes Project Phase 3 reference panel (referred to as 1000G), representing the general human population without ascertainment for ASD or any neurodevelopmental condition. The same 55 genes were queried for rare missense variants, filtered, and annotated under identical criteria. The 1000G dataset serves as the control against which SFARI case variant burden is evaluated.

For each gene in each cohort, the following quantities were recorded: chromosomal coordinates; coding sequence length, L, in base pairs; the number of matched variants, N, with available REVEL scores; the sum of REVEL scores across all matched variants; and three formula scores described in the next sections. It is important to note that the 1000G cohort consistently yields more matched variants per gene than SPEC, reflecting the larger size of the reference panel rather than a biological difference. This asymmetry is addressed by the per-variant normalization described in the following sections.

Within the selected gene intervals, 2,709,082 variants were retrieved from the 1000G cohort and 2,225,591 from the SPEC cohort. Variants were retained if they had a non-zero allele frequency in the cohort under analysis and carried a REVEL annotation. Because REVEL is defined only for missense substitutions, the majority of retrieved variants fall outside of its scope: 26,584 variants (1.0%) were retained in 1000G and 14,057 (0.6%) in SPEC, with the excluded fraction consisting predominantly of non-coding, intronic, and synonymous variants. This restriction to missense variation is an inherent property of the current formulation and is discussed further in Section 4.

### 2.4. The Composite Pathogenicity Formula

The formula is related to the Hardy–Weinberg principle. Let H denote the expected heterozygosity at a biallelic locus. Then,

(1) H=2p(1−p)

Here, p is the population-level minor allele frequency (AF) of the variant. The quantity in Equation (1) has a direct biological interpretation: it represents the proportion of individuals in the population who carry one copy of the variant and are therefore potentially exposed to the functional effect of the variant in a haploinsufficient gene. Variants with very low frequency (p → 0) contribute minimally to 2p(1−p), while variants at intermediate frequencies contribute maximally. For rare disease variants, the term up-weights the more common ones.

This weighting alone, however, does not distinguish a rare benign variant from a rare damaging one. Two variants with identical allele frequencies will receive identical Hardy–Weinberg weights regardless of their predicted functional impact. The REVEL score is introduced as a multiplicative pathogenicity filter: each variant’s Hardy–Weinberg weight is scaled by its REVEL score, so that rare variants predicted to be benign contribute little to the gene-level score regardless of their frequency, while rare variants predicted to be damaging contribute proportionally to their predicted impact.

H is computed from each cohort’s own allele frequencies, so the same gene receives different scores in cases and controls. That is what enables contrast between cases and controls, and it is exactly what pLI, LOEUF, and MTR cannot do, being fixed population-derived constants. Only using allele frequency is unrepresentative of diploid carrier exposure, as it is linear; other options, such as −logp, are unbounded. H is bounded in the interval [0, 0.5], continuous, threshold-free, and needs only the allele frequency present in any VCF. Limitations of H are related to the fact that it increases monotonically with p, so more common variants contribute more; it assumes Hardy–Weinberg equilibrium, which may be violated under selection or population structure; and it is sensitive to cohort ascertainment, since p is estimated from the cohort.

The product 2p(1−p) × REVEL is then summed across all N variants in the gene with available *REVEL* scores. This produces a raw gene-level pathogenicity score. To remove the gene-length confound described in the Introduction, the sum is divided by the coding sequence length, L, yielding a length-normalised composite score. The full formula is therefore defined as Equation (2):

(2) $$P_{f}=\frac{1}{L}\sum_{i}2p_{i}(1-p_{i})\times REVEL_{i}$$

where the sum runs over all N variants in the gene with available *REVEL* scores, $p_{i}$ is the allele frequency of variant i in the relevant cohort, $REVEL_{i}$ is its *REVEL* score, and L is the coding sequence length in base pairs.

To evaluate the independent contribution of each component, two reduced formulas were computed alongside the full formula for every gene in both cohorts. The first reduced formula retains the allele frequency and length components but removes *REVEL*:

(3) $$P_{af}=\frac{1}{L}\sum_{i}2p_{i}(1-p_{i})$$

Equation (3) measures rare variant burden weighted by heterozygosity and normalized by gene length, but it treats all variants as equally pathogenic regardless of their predicted functional impact. It is equivalent to a length-normalized heterozygosity burden score. The second reduced formula retains only the REVEL component:

(4) $$P_{r}=\frac{1}{N}\sum_{i}REVEL_{i}$$

Equation (4) measures the average predicted pathogenicity of variants in the gene without any weighting by allele frequency or correction for gene length. It is the closest analogue to simply summarizing *REVEL* scores at the gene level, as might be done naively before the present framework was introduced. The three formulas form a nested comparison: $P_{r}$ contains only pathogenicity prediction, $P_{af}$ contains only population genetics context, and $P_{f}$ integrates both. Comparing their performance on the same data isolates each component’s contribution.

Several properties of $P_{f}$ are worth noting explicitly. First, the formula is additive across variants: each variant contributes independently to the gene score, and the total score increases with the number of rare damaging variants in the gene. This means that a gene with many moderate-*REVEL* variants can score similarly to a gene with few high-*REVEL* variants if their allele frequencies are comparable, a property that is biologically reasonable but becomes a limitation when distributional information matters. This motivates the nonlinear extension described in the Future Directions section.

Second, the formula is cohort-specific: allele frequencies, p_i_, are taken from the cohort being scored, so $P_{f}(SPEC)$ and $P_{f}(1000G)$ may differ even for the same variant if its observed frequency differs between the two datasets. This is intentional and is what allows the formula to detect cohort-level enrichment.

Third, the formula requires *REVEL* scores, which are only defined for missense variants. Variants without *REVEL* annotations are excluded from the sum. In datasets where many variants lack *REVEL* scores, such as ancient DNA panels targeting population ancestry markers rather than clinical variants, this exclusion can substantially reduce N and introduce ascertainment bias. This limitation is discussed in the last section.

Raw formula scores are not directly comparable between the SPEC and 1000G cohorts because the two datasets contain different numbers of matched variants per gene. A gene with 150 SPEC variants and 350 1000G variants will produce a higher raw score in 1000G by virtue of sample size alone, even if SPEC variants are individually more pathogenic. To remove this sampling depth effect, all scores are converted to per-variant scores (PVS) by dividing by the matched variant count, N, shown in Equation (5):

(5) $$PVS=\frac{P_{\left\{f,r,af\right\}}}{N}$$

$Log_{2}$ enrichment is then defined as follows:

(6) $$log_{2}\;enrichment=\log_{2}\frac{PVS\left(SPEC\right)}{PVS\left(1000G\right)}$$

A positive $log_{2}$ enrichment in Equation (6) indicates that SFARI case variants score higher per variant than general-population control variants in the same gene: the expected direction if the gene genuinely contributes to ASD pathogenicity. A value of zero indicates no difference. A negative value indicates the control cohort scores higher, which may reflect formula limitations, data artifacts, or genuine biological complexity. The $log_{2}$ scale is used throughout because it is symmetric around zero.

### 2.5. Statistical Comparison of the Three Formulas

For each of the three scoring schemes, $log_{2}$ enrichment values were computed for all 55 genes, producing three paired vectors of length 55. The performance of the full formula relative to each reduced formula was assessed using three complementary statistical approaches.

A paired Student’s *t*-test was used to test whether the mean $log_{2}$ enrichment was significantly higher under the full formula than under each comparator. The paired design is appropriate because the same 55 genes are scored under both formulas, removing between-gene variance from the comparison.

A Wilcoxon signed-rank test was computed as a non-parametric complement, making no assumption about the distribution of $log_{2}$ enrichment differences. This is important because the enrichment distribution across genes is not guaranteed to be Gaussian, particularly given the presence of outlier genes such as KMT2C and MYT1L at the extremes of the distribution.

Cohen’s d for paired differences is defined in Equation (7) as the effect size measure:

(7) $$d=\frac{\mu (∆)}{\sigma (∆)\;},$$

where μ is the mean of ∆, σ is the standard deviation, and ∆ is defined as follows:

(8) $${∆}_{i}=\log_{2}\left(\frac{P_{f}^{SPEC}\left(i\right)}{P_{f}^{1000G}\left(i\right)}\right)-\;\log_{2}\left(\frac{P_{q}^{SPEC}\left(i\right)}{P_{q}^{1000G}\left(i\right)}\right)$$

Here, $P_{f}^{SPEC}(i)$ and $P_{\left\{af,r\right\}}^{SPEC}(i)$ are the per-variant scores of gene i in the SPEC cohort; $P_{\left\{af,r\right\}}^{1000G}\left(i\right)$ and $P_{f}^{1000G}\left(i\right)$ are the corresponding per-variant scores in the 1000G cohort; μ(∆) is the mean of ∆ across all 55 genes; and σ(∆) is the standard deviation of ∆ across all 55 genes, q∈{af,r}. Values of d ≈ 0.2, 0.5, and 0.8 correspond to small, medium, and large effects, respectively, by conventional benchmarks. All statistical analyses were performed in Python 3 (version 3.14.5) using *pandas* (version 3.0.3), *numpy* (version 2.4.6), and *scipy.stats* (version 1.17.1) libraries.

To assess whether the aggregate results were driven by a small number of influential genes rather than a consistent effect across the gene set, confidence intervals were estimated by bootstrap resampling [24]. The 55 genes were resampled with replacement to form 10,000 bootstrap replicates, and for each replicate, the aggregate statistics, the mean $log_{2}$ enrichment under each formula, the fraction of genes with positive enrichment, the fraction of genes in which the full formula exceeded each reduced formula, and the paired Cohen’s d for each comparison were recomputed. Ninety-five percent confidence intervals were taken as the 2.5th and 97.5th percentiles of the resulting bootstrap distributions. It should be noted that this procedure resamples across genes rather than across variants within a gene, because the dataset provides gene-level aggregates rather than individual variant scores. The across-gene bootstrap therefore addresses whether the reported aggregate statistics are stable to the particular composition of the gene set, which is the relevant robustness concern at this level of analysis.

The paired *t*-test is applied to the mean of 55 paired per-gene differences. By the Central Limit Theorem, the sampling distribution of a mean over 55 paired observations is approximately normal irrespective of the shape of the underlying per-gene distribution, and the log_2_ transformation additionally renders the enrichment values approximately symmetric. Parametric testing is therefore appropriate here. Because the reported conclusions do not rest on the parametric assumption alone, every comparison is accompanied by a Wilcoxon signed-rank test, which makes no distributional assumption, and by the label-permutation procedure described below, which yields a fully distribution-free empirical significance estimate. All three approaches appear to agree.

As an internal negative control that requires no external gene set, a label-permutation test [25] was performed to assess whether the observed enrichment could be due to chance alone. In each of 10,000 permutations, the assignment of the case (SFARI) and control (1000G) roles was randomly swapped independently for each gene, and the mean $log_{2}$ enrichment and the fraction of genes with positive enrichment were recomputed under the permuted labeling. These permuted values form a null distribution against which the observed statistics were compared. An empirical one-sided *p*-value was computed as the proportion of permutations in which the permuted statistic was equal to or exceeded the observed value. Under this null, in which case–control labels carry no information, the mean $log_{2}$ enrichment is expected to center on zero, and the fraction of positive genes is one half; an observed value lying far in the tail of the null distribution indicates that the enrichment reflects a genuine difference between cohorts rather than an artifact of the scoring construction.

### 2.6. Dynamic Range Analysis

Beyond aggregate statistical comparison, the dynamic range of each formula across the 55 genes is examined. Dynamic range here refers to the spread of $log_{2}$ enrichment values across genes: a formula with high dynamic range clearly separates high-burden genes from low-burden genes, while a formula with low dynamic range compresses all genes toward a similar score and provides little discriminative power for ranking. Dynamic range was assessed by computing, for each formula, the spread of $log_{2}$ enrichment values across the 55 genes. Two measures of spread were used: the standard deviation, capturing the typical dispersion around the mean, and the total range (maximum minus minimum), capturing the separation between the most and least-enriched genes.

### 2.7. Experimental Setup and Hardware

A Lenovo^®^ ThinkPad (Lenovo, Hong Kong, China) with an Intel^®^ Core i5 CPU (Intel, Santa Clara, CA, USA) and 16 GB of RAM is used to process and analyze the genes.

## 3. Results

### 3.1. Normalization Properties

**Figure 2:**
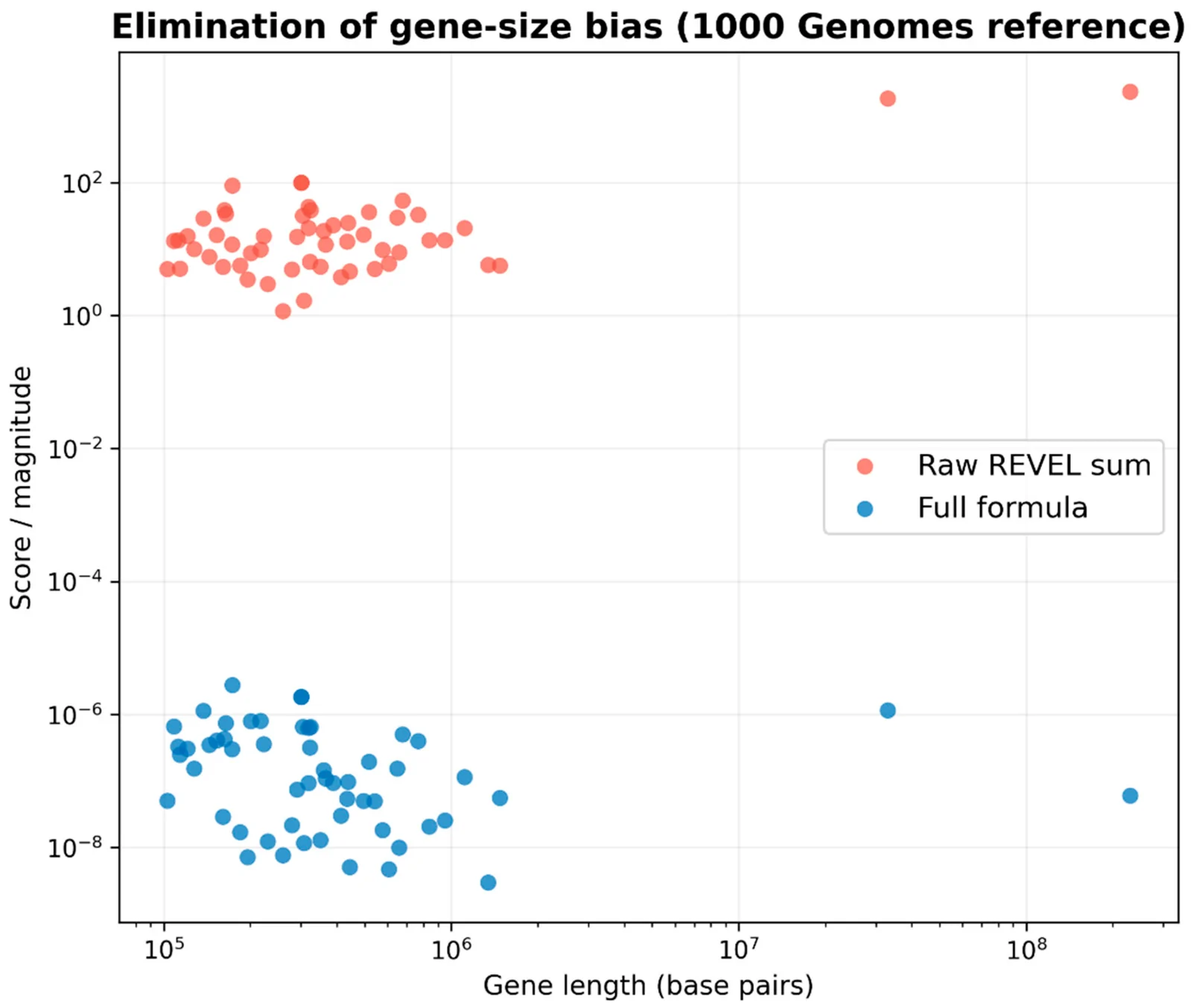
Elimination of gene-length bias, computed on the 1000G cohort. Both the X- and Y-axes are logarithmically scaled. The blue points indicate the score for the full formula, while the red dots show only the *REVEL* component.

Before evaluating biological signals, we confirmed that the composite formula removes the two structural confounds identified in the Introduction: gene length and variant count. Figure 2 shows, for the 1000G reference cohort, the raw *REVEL* sum and the full composite score against gene coding length on logarithmic axes. The raw *REVEL* sum exhibits a clear positive trend with gene length: longer genes accumulate more missense variants and, therefore, larger *REVEL* sums, independent of any difference in per-variant pathogenicity. This is the size artifact that makes naive *REVEL* summation unsuitable for cross-gene comparison. After length normalization, the full composite score shows no such trend, occupying a narrow band across three orders of magnitude in gene length. The formula therefore measures pathogenic burden per unit of coding sequence rather than gene size.

Each point represents one of the 55 genes, plotted twice-once for its raw *REVEL* sum and once for its composite score, so that the two quantities can be compared directly across the same length axis.

This finding is confirmed quantitatively. On the logarithmic scale of Figure 2, the raw *REVEL* sum correlates strongly with gene length in both cohorts ($Pearson\;r\;=\;0.61,\;p\;=\;7\times 10^{-7}$ in 1000G; $r\;=\;0.59,\;p\;=\;2\times 10^{-6}$ in SPEC), whereas the composite score shows no significant association (r = −0.15, p = 0.28 and r = −0.12, p = 0.37 respectively). Rank-based correlations show the same removal of the positive trend, with a small residual negative association for the composite score (Spearman ρ = −0.33, p = 0.015 in 1000G), indicating that division by full gene length slightly overcorrects the bias. We note this as a minor limitation of the current normalization.

### 3.2. Cohort-Level Performance Across All 55 Genes

**Figure 3:**
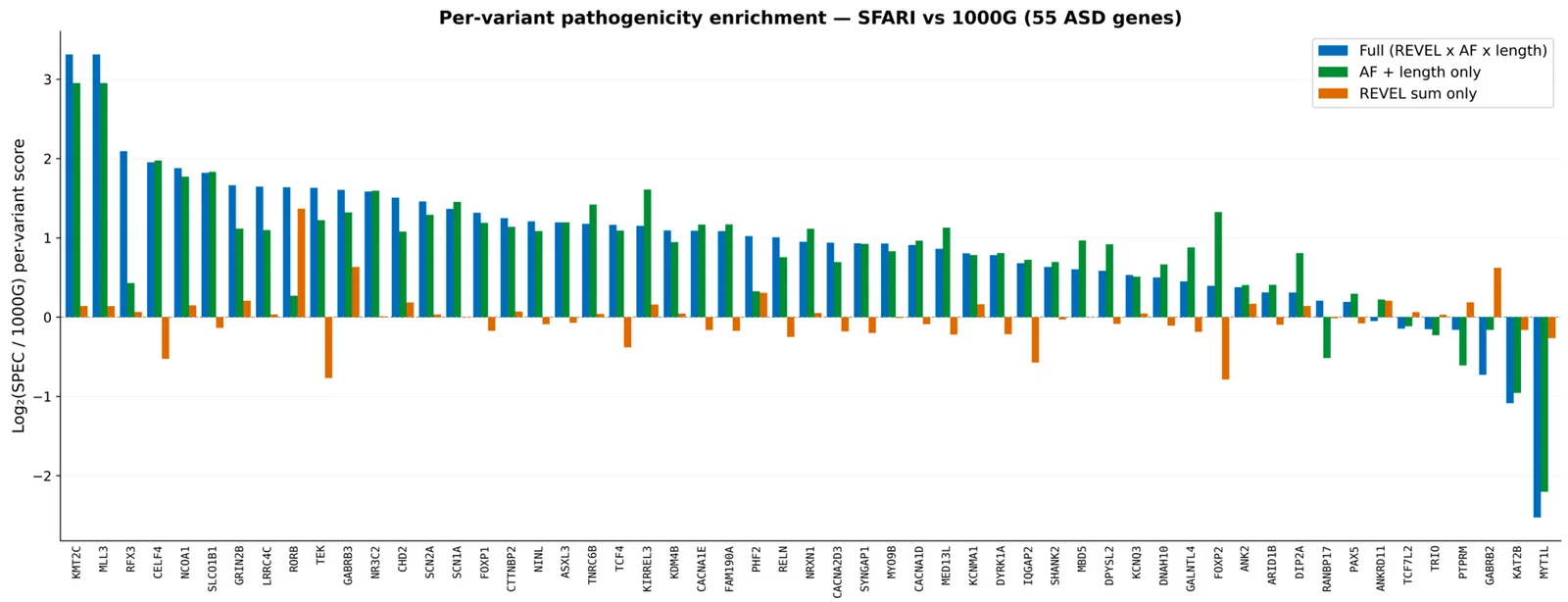
Each bar is the log_2_ enrichment defined in Equation (6). Zero indicates identical scoring in the two cohorts, positive values indicate higher per-variant burden in the case cohort, and negative values indicate that the population reference scores higher, which arises when the control variants in that gene are on average more frequent, more damaging, or both, than those observed in cases. Negative values are not biologically anomalous as they identify genes showing no excess missense burden in cases under this metric, which may reflect pathogenic mechanisms outside the scope of *REVEL*, small case-variant counts, or a genuine absence of missense enrichment.

Having established that the formula removes structural artifacts, we evaluated its ability to discriminate the SFARI case cohort from the 1000G population reference. For each gene, the per-variant score was computed in both cohorts and expressed as a $log_{2}$ enrichment, with positive values indicating higher per-variant pathogenicity in the SFARI cohort. Figure 3 shows the $log_{2}$ enrichment for all 55 genes under the three formulas, sorted by the full-formula value. Under the full formula, 48 of 55 genes (87.3%) show positive enrichment, with a mean per-variant score ratio of 2.16 and a mean $log_{2}$ enrichment of 0.91. The AF-and-length formula produces a similar mean $log_{2}$ enrichment of 0.85, while the *REVEL*-only formula produces a mean $log_{2}$ enrichment close to zero, with only 26 of 55 genes positive: no better than chance. This ordering is the central empirical result: at the gene level, the population-genetics component carries most of the discriminating signal, the *REVEL* component alone carries almost none, and the two combined produce the strongest and most consistent separation.

**Figure 4:**
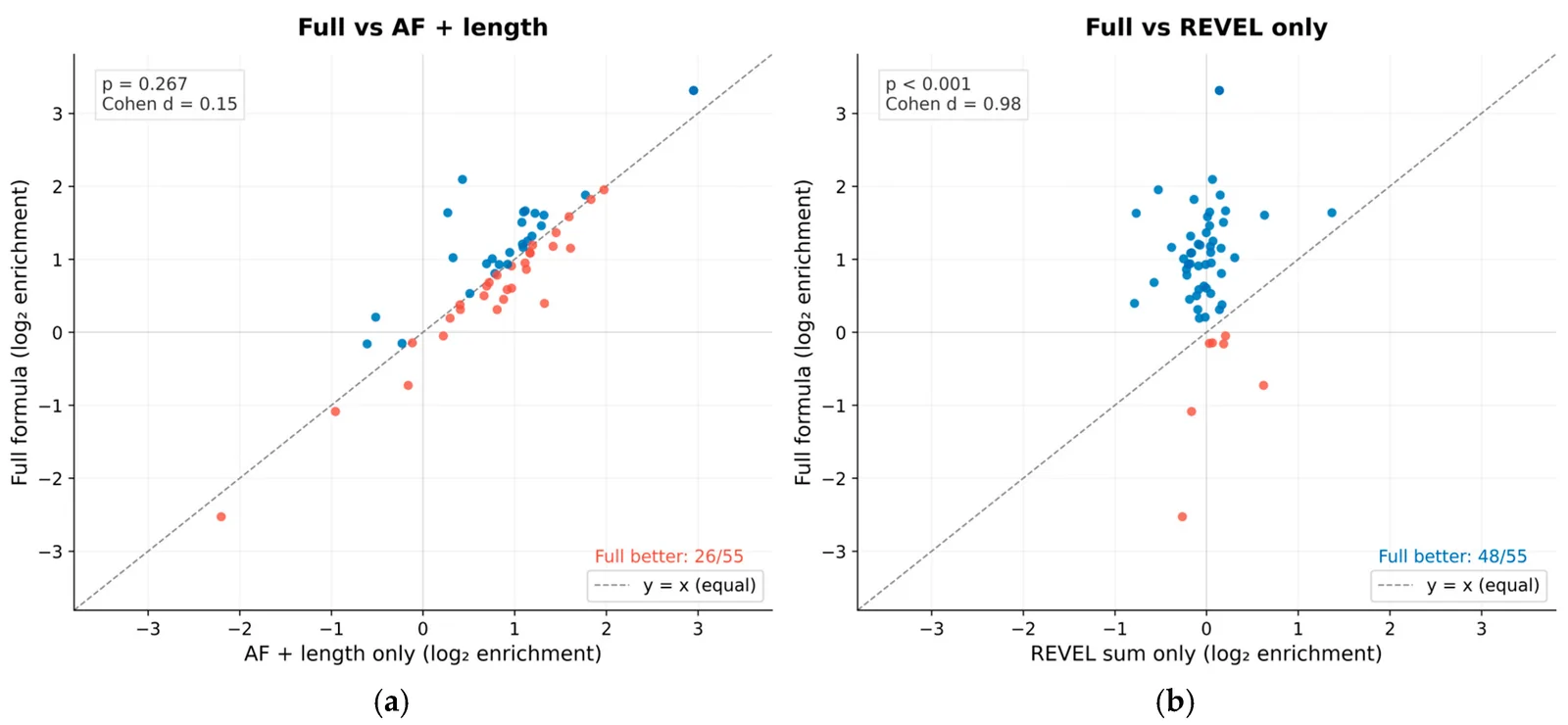
Two scatter panels comparing the full formula against each reduced formula: (**a**) full versus AF + length and (**b**) full versus *REVEL*-only. Each point is one gene; the horizontal axis gives its log_2_ enrichment under the comparator formula and the vertical axis its log_2_ enrichment under the full formula, so points above the dashed y = x diagonal are genes for which the full formula yields greater case–control separation. Annotations report the paired *t*-test *p*-value, Cohen’s d, and the number of genes favoring the full formula. In panel (**a**), the points cluster tightly around the diagonal, reflecting the modest and non-significant aggregate advantage over AF + length, with RFX3 and RORB as exceptions; in panel (**b**), nearly all points lie well above the diagonal, reflecting the large advantage over *REVEL*-only aggregation.

The pairwise advantage of the full formula over each reduced formula was quantified using paired tests across the 55 genes (Figure 4). Each point represents a gene; points above the diagonal indicate genes for which the full formula yields higher enrichment than the comparator. Compared to the *REVEL*-only formula, the full formula is superior in 48 of 55 genes, with a mean $log_{2}$ difference of 0.93, a large effect size (Cohen’s d = 0.98), and high statistical significance (paired *t*-test p < 0.0001; Wilcoxon p < 0.0001). The mechanistic explanation is that summing *REVEL* scores without allele-frequency weighting cannot distinguish a gene carrying many common, low-impact variants as population-reference genes typically do: from one carrying a few rare, high-impact variants. The allele-frequency term supplies exactly this distinction. Compared to the AF-and-length formula, the improvement is directional but modest in aggregate: the full formula is higher in roughly half the genes, with a small mean $log_{2}$ difference of 0.06 that does not reach significance (paired *t*-test p = 0.27; Cohen’s d = 0.15). We interpret this as an accurate description of where the *REVEL* term matters.

### 3.3. Dynamic Range

**Figure 5:**
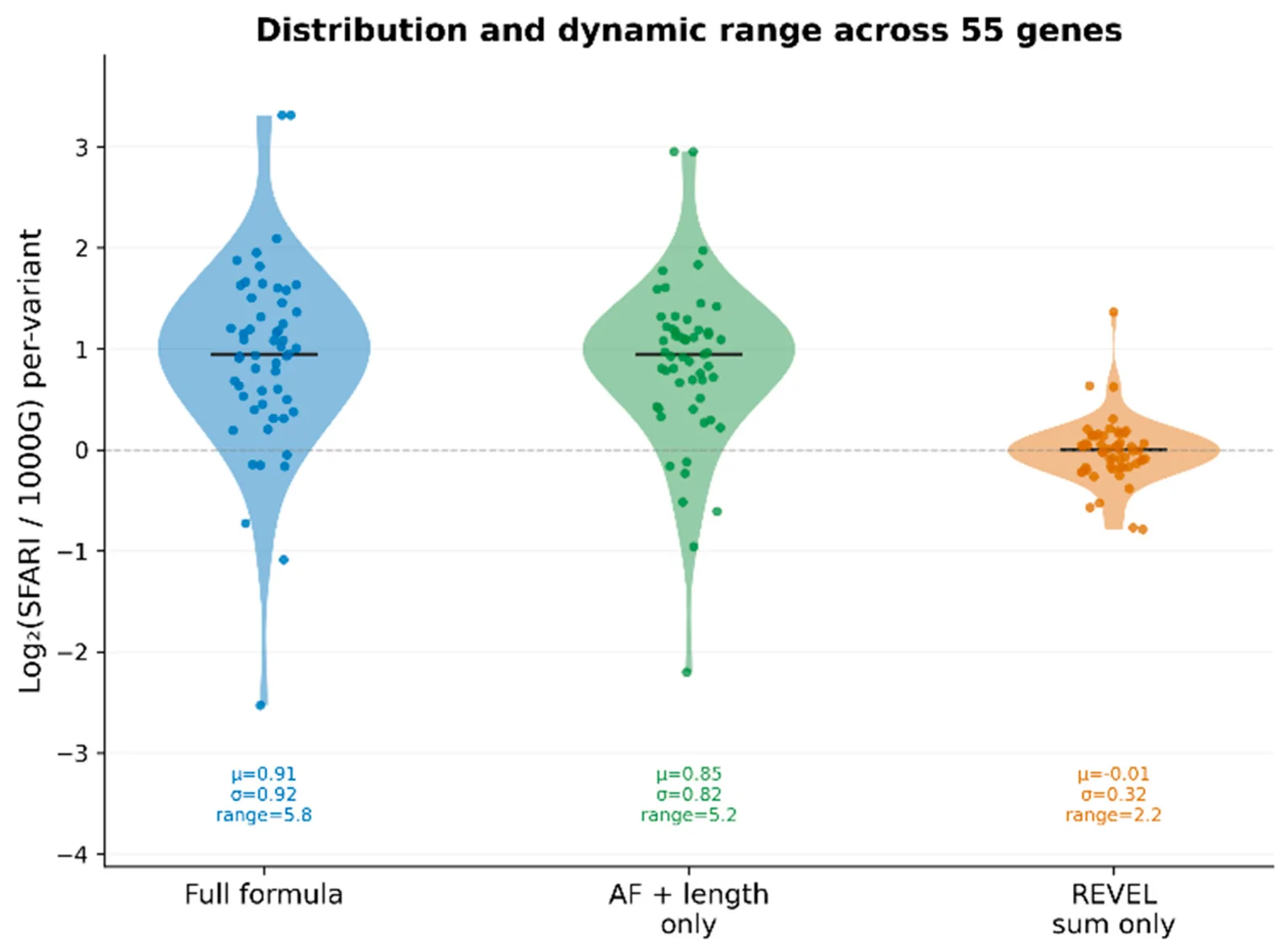
Distribution and dynamic range across 55 genes. The mean (μ), standard deviation (σ), and total range are annotated: the full formula spans more than five log_2_ units, whereas the *REVEL*-only formula compresses all genes into a narrow band around zero and therefore provides little discriminative power for ranking. Colored dots indicate genes.

Aggregate means do not fully capture the usefulness of a formula for ranking genes. A formula that compresses all genes toward a single value provides little discriminative power even if its mean enrichment is positive. We therefore characterized the dynamic range of each formula as the spread of $log_{2}$ enrichment values across the 55 genes (Figure 5).

Each point in Figure 5 is one gene, with the outline showing the kernel density of the 55 values and the horizontal bar marking the median. The full formula produces the widest spread, separating high-burden genes such as KMT2C ($log_{2}\approx 3.3$) from low-burden genes such as MYT1L ($log_{2}\approx -2.5$) across more than five $log_{2}$ units. The *REVEL*-only formula compresses all genes into a narrow band around zero. The expanded dynamic range of the full formula is what makes it suitable for ranking genes within a set and, ultimately, for comparing aggregate burden across gene sets, which is the intended application of the metric.

### 3.4. Statistical Tests

**Figure 6:**
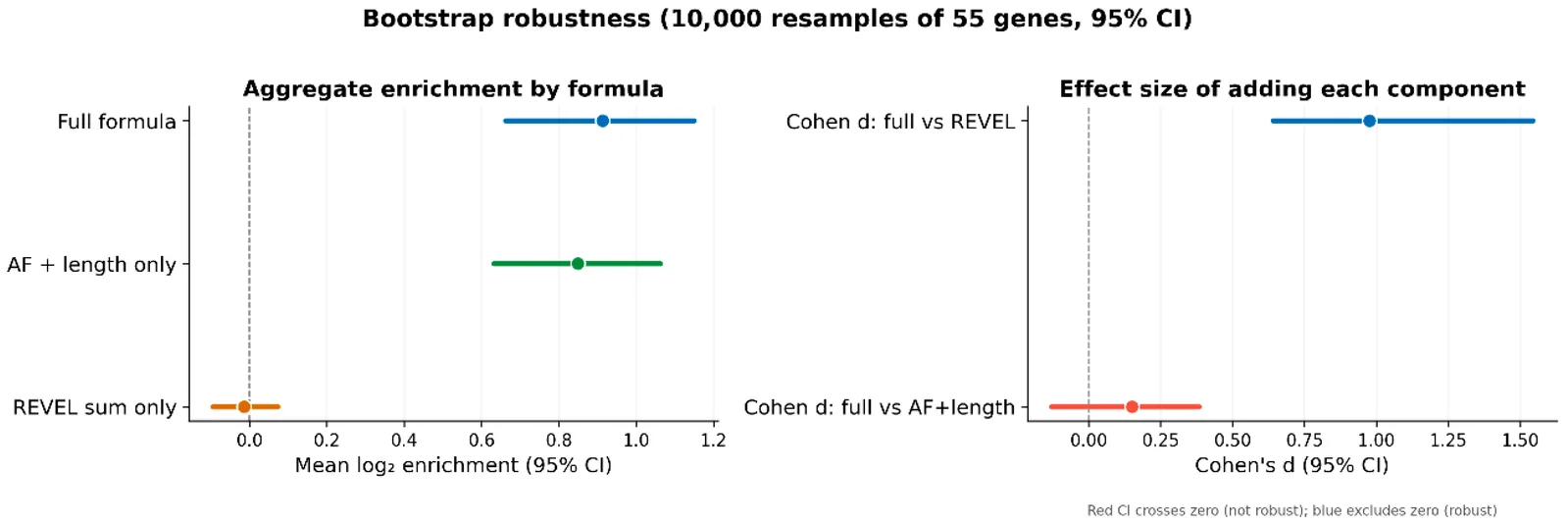
Bootstrap CI on the selected 55 genes. **Left panel**: Mean $log_{2}$ enrichment of SFARI relative to 1000G under each of the three formulas (full, allele frequency plus length, and *REVEL* sum only); the vertical dashed line marks zero enrichment, and intervals lying entirely to the right of it indicate enrichment that is stable across resampling. **Right panel**: Paired effect size (Cohen’s d) for the two key comparisons, the full formula against *REVEL*-only and the full formula against allele frequency plus length; the vertical dashed line marks an effect size of zero. Intervals are coloured blue where they exclude zero (a robust effect) and red where they cross zero (an effect that is not robust). Dashed vertical line indicates the origin of both values.

Two analyses were performed to test the stability and internal validity of the results reported above. The bootstrap analysis quantifies the stability of the headline statistics (Figure 6). The mean $log_{2}$ enrichment under the full formula was 0.914, with a 95% confidence interval of [0.66, 1.15] that excludes zero, confirming that the enrichment is not driven by a handful of outlier genes. The proportion of genes with positive enrichment, 48 of 55, was stable with a confidence interval of [0.78, 0.95]. The advantage of the full formula over the *REVEL*-only formula was robust, with a Cohen’s *d* confidence interval of [0.64, 1.54] that excludes zero, and the full formula exceeded the *REVEL*-only formula in a stable 87% of genes (confidence interval [0.78, 0.95]). In contrast, and consistent with the non-significant paired test reported above, the effect size for the comparison between the full formula and the allele-frequency-and-length formula had a confidence interval of [−0.13, 0.38] that includes zero, confirming that the incremental contribution of the *REVEL* term over allele frequency and length alone is not robust in aggregate across the full gene set.

**Figure 7:**
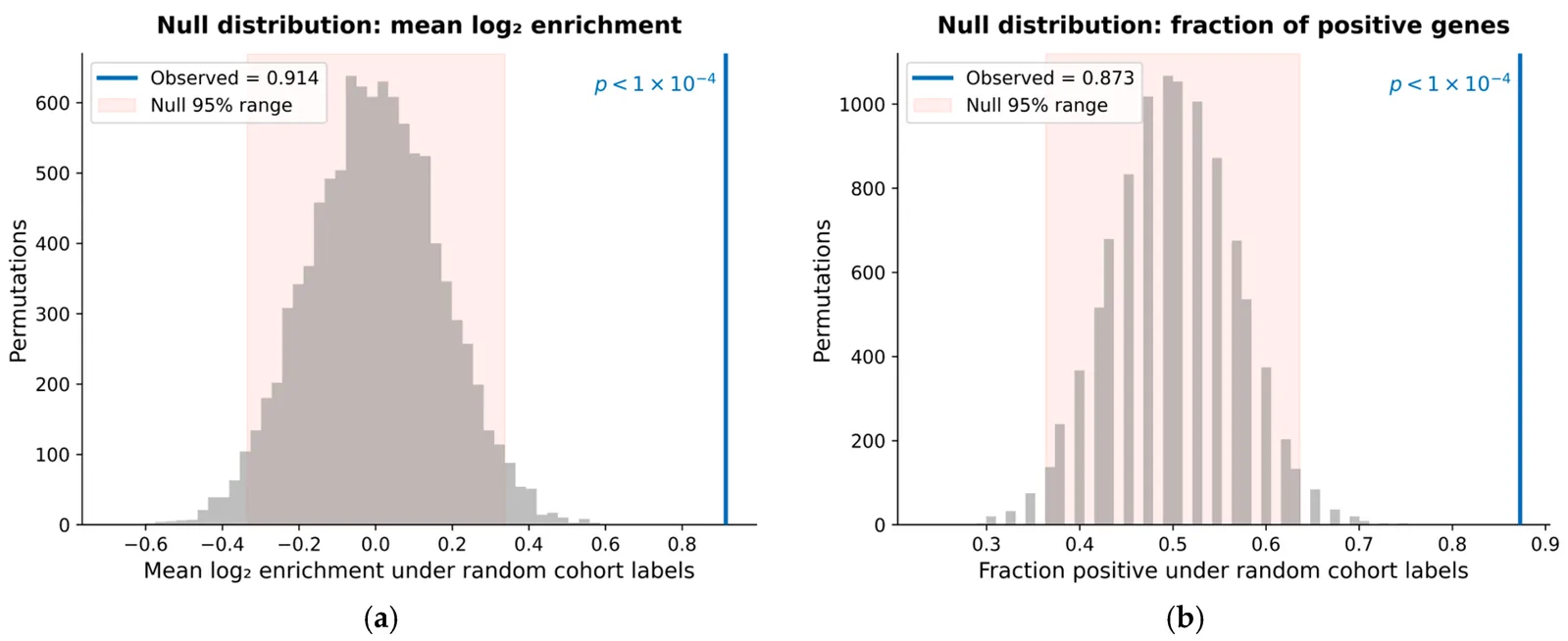
Permutation tests for negative control. Grey histograms show the distribution of each statistic under the permuted (null) labeling; the shaded band marks the central 95% of the null distribution. The solid vertical line marks the value actually observed in the correctly labeled data. Left panel (**a**) mean $log_{2}$ enrichment across the 55 genes, with a null distribution centered near zero as expected when labels carry no information. Right panel (**b**) the fraction of genes showing positive enrichment, with a null distribution centered near one half.

The label-permutation test provides the more fundamental check. When the case and control labels were randomly swapped per gene across 10,000 permutations, the mean $log_{2}$ enrichment in the null distribution was approximately zero, and the null fraction of positive genes was approximately one half, as expected when the labels carry no information. The observed mean $log_{2}$ enrichment of 0.914 and observed fraction of positive genes of 0.873 both fell far outside the corresponding null 95% ranges of approximately [−0.34, 0.34] and [0.36, 0.64], respectively, with empirical significance below the resolution of the permutation procedure in both cases ($p\;<\;1\times 10^{-4}$; zero of 10,000 permutations reached the observed value) (Figure 7). The observed enrichment therefore cannot be attributed to the structure of the formula acting on randomly labeled data, and reflects a genuine difference between the SFARI and 1000G cohorts.

### 3.5. Further Results

The application of the metric can be illustrated by tracing the calculation through several representative genes. For each gene, the product, 2p(1−p)·REVEL, is summed across all annotated variants in the gene interval, divided by coding-sequence length to give $P_{f}$, and divided by the variant count to give the per-variant score, whose ratio between cohorts is the $log_{2}$ enrichment plotted in Figure 3, Figure 4 and Figure 5:

- KMT2C illustrates the high-burden case: its 149 case variants yield a summed weighted score of 2.24 against 0.55 from 362 population variants, so the case cohort produces a four-fold larger sum from fewer than half as many variants, giving an approximately ten-fold per-variant enrichment ($log_{2}\;=\;3.31$).
- RFX3 shows where the *REVEL* term is decisive, raising an enrichment of only 1.35-fold under allele frequency and length alone to 4.27-fold ($log_{2}\;=\;2.09$), because its case variants carry substantially higher predicted pathogenicity at comparable allele frequencies.
- CHD2 and SCN2A represent the typical behavior of the panel, with moderate enrichment ($log_{2}\;=\;1.51\;and\;1.46$, respectively) and all three formulas agreeing in direction.
- MYT1L illustrates the opposite outcome: nine case variants sum to 0.002 against 0.027 from 25 population variants, giving a per-variant score roughly six-fold lower in cases $(log_{2}\;=\;-2.53$). As noted in Section 4, such genes are flagged by the metric as candidates for investigation by other means rather than dismissed.

## 4. Discussion and Conclusions

This work introduced a composite gene-level pathogenicity metric that integrates Hardy–Weinberg expected heterozygosity, coding sequence length, and per-variant *REVEL* scores, and evaluated it against two reduced formulas across 55 high-confidence ASD genes derived from three large-scale studies in a case cohort and a population reference. Three findings emerge consistently from the analysis.

First, the composite formula removes structural confounds that render naive *REVEL* summation unsuitable for cross-gene comparison. Length normalization eliminates the artificial correlation between raw *REVEL* sum and gene size, and the allele-frequency weighting decouples the score from variant count, so that the metric reflects pathogenic burden per unit of coding sequence rather than gene size or sequencing depth.

Second, at the cohort level, the full formula discriminates SFARI case variants from population controls in 48 of 55 genes, with a mean per-variant enrichment of 2.16-fold. The decomposition into reduced formulas is informative in itself: the allele-frequency and length component carry most of the discriminating signal, the *REVEL* component in isolation carries almost none, and the two combined produce the strongest and most consistent separation. The advantage of the full formula over naive gene-level aggregation of *REVEL* scores is large and statistically significant (Cohen’s d = 0.98, p < 0.0001). We emphasize that this comparison concerns gene-level aggregation strategies and is not a claim about *REVEL*’s performance as a variant-level pathogenicity predictor, for which it remains a well-validated tool.

Third, the full formula has the widest dynamic range of the three, separating high-burden from low-burden genes across more than five $log_{2}$ units. This property, rather than the aggregate mean enrichment, is what makes the metric useful for ranking genes within a set and for the eventual application to whole gene sets.

The 55 genes analyzed here were drawn from the high-confidence ASD genes reported across three large studies, which prioritize genes with robust, replicated evidence of disease association and therefore provide a well-characterized positive control against which formula behavior can be judged.

It is useful to position the proposed metric relative to existing gene-level measures. MTR quantifies regional missense constraint by comparing observed to expected missense variation within a sliding window, and is estimated once from a large population reference. LOEUF, and its predecessor, pLI, summarize a gene’s tolerance to loss-of-function variation from gnomAD. Gene-level summaries derived from AlphaMissense [26] aggregate per-variant pathogenicity predictions across a transcript, and therefore share the structure of the *REVEL*-only comparator evaluated here. What these approaches have in common is that they yield a single fixed value per gene, estimated from a reference population. They are informative priors about a gene’s constraint or predicted deleteriousness, but they cannot express a difference between two cohorts, because they are not functions of a cohort’s own allele frequencies. The metric proposed here is computed separately within each cohort from that cohort’s observed frequencies, so the same gene receives different scores in cases and controls, and the contrast between them is the quantity of interest. The two classes of measure are therefore complementary rather than competing: constraint metrics describe what a gene is, whereas the present metric describes what a gene looks like in a particular sample.

Several limitations affect the conclusions of this study, and each maps onto a particular direction for development. The most fundamental is that *REVEL* is defined only for missense variants. Non-coding variants in promoters, enhancers, and untranslated regions; synonymous variants that disrupt splicing; and copy-number and structural variants are all invisible to the current formula. In genes whose principal pathogenic mechanism is non-coding, the composite score will systematically underestimate disease burden. Because the longer-term goal involves whole-genome sequence data, in which non-coding variants are the majority, this limitation is significant rather than marginal. A second limitation concerns the aggregation of variant information. The present dataset provides gene-level sums of *REVEL* scores together with variant counts, rather than individual variant-level scores. Summation conflates distinct pathogenic profiles: a single variant with a *REVEL* score of 0.95 and five variants, each with a score of 0.19, both produce the same sum, yet represent very different biological situations. The additive, linear structure of the current formula cannot distinguish these cases. Thus, normalizing by the number of scored variants introduces its own distortion, because a gene represented by a single low-scoring variant can receive an inflated per-variant score relative to a gene represented by many variants. These constraints have direct implications for interpretation. Because the underlying variant records are not retained, it cannot be established whether a gene’s score reflects a single influential variant or a broad distribution across many, and neither variant-level quality control nor stratification by variant class is possible after aggregation. The same constraint restricts the resampling procedure to the across-gene level, and precludes implementation of the nonlinear reweighting proposed below, which is a function of each individual score. Access to individual-level variant data is therefore a prerequisite for the next stage of this work and would simultaneously address several of the limitations described here. A third limitation is the imbalance in variant counts between cohorts. The population reference systematically contains more matched variants per gene than the case cohort, reflecting differences in sample size and sequencing strategy rather than biology. Per-variant normalization mitigates this but does not equalize statistical power across genes, and genes represented by very few case variants are strongly influenced by the properties of individual variants.

Two further limitations of our metric are practical in nature. The gene coordinates used here are defined in the GRCh37/hg19 assembly, whereas the large-scale resources targeted for the next phase, including gnomAD v4 and the CCDG (Centers for Common Disease Genomics) whole-genome data, are defined in the GRCh38 assembly; accurate integration will require recalibration of gene boundaries before variant extraction. Finally, the metric has been evaluated gene by gene so far. Its intended application is to quantify aggregate burden across functionally defined gene sets, and that gene-set behavior has not yet been tested.

A further limitation concerns the composition of the gene panel itself. The panel was assembled as the union of three published ASD gene lists rather than their intersection, and therefore represents genes reported as ASD-associated by at least one study rather than a consensus high-confidence set; of the 55 entries, only eight appear in all three source studies. More consequentially, the requirement that a gene carry more than 50 retrieved variants introduces an ascertainment bias whose direction is opposite to what a pathogenicity metric would ideally have. Genes under strong purifying selection accumulate fewer variants in population data precisely because deleterious variation is removed, so a variant-count threshold preferentially retains mutation-tolerant genes and excludes the most constrained ones. Several of the most strongly established ASD genes, including CHD8, SHANK3, PTEN, ADNP, POGZ, and TBR1, are consequently absent from the analysis despite appearing in all three source studies. The panel evaluated here should therefore be understood as ASD-associated genes with sufficient variant coverage for stable estimation, and the metric’s behavior on highly constrained, variant-sparse genes remains untested. Relaxing this threshold, at the cost of noisier per-gene estimates, is a necessary step in future work.

Finally, the metric has been evaluated on a single disease context. The ASD gene set serves here as a pilot application, and the method has not yet been externally validated in independent disease cohorts; such validation is a necessary step before general applicability can be claimed.

The limitations above define a clear development path, and the central planned extension is a move from the present linear formula to a nonlinear composite metric that incorporates gene context. The motivation is that biological pathogenicity rarely scales linearly with a predictor such as *REVEL*. A linear weighting treats a change from 0.4 to 0.5 as equivalent to a change from 0.8 to 0.9, whereas, in practice, pathogenicity often follows a threshold or sigmoidal relationship in which effects escalate sharply beyond a critical value. The current hard filter, which retains only variants above a fixed *REVEL* cutoff, converts this into an abrupt step function and discards information from moderate-effect variants that may act cumulatively. Replacing the linear *REVEL* weight with a sigmoidal transformation of the form 1 / (1 + exp(−γ(REVEL − θ))) would suppress low-score noise smoothly while amplifying variants approaching the high-confidence range, with θ setting the threshold and γ controlling its sharpness, without the information loss of a hard cutoff.

The permutation and bootstrap analyses together establish the internal validity and stability of the reported results: the enrichment signal is not an artifact of the scoring construction, and the headline statistics are robust to the composition of the gene set. These analyses do not, however, establish external specificity, namely whether the formula scores known autism genes above genes with no expected disease relevance. Addressing this requires extending the variant-calling pipeline, which operates on raw VCF files, to a control gene set, and is therefore grouped with the evolutionary three-cohort analysis as the principal direction for subsequent work. We regard the label-permutation procedure as a methodological contribution in its own right, as it provides a self-contained internal negative control that requires no additional data and is generally applicable to gene-level cohort-comparison metrics of this kind.

The second component of the planned reformulation introduces gene-level evolutionary context, which the current formula lacks entirely. A *REVEL* score of 0.6 in a gene under intense purifying selection should not carry the same weight as the same score in a mutation-tolerant gene. Weighting each variant by a factor derived from the gene’s loss-of-function intolerance, such as (1 − LOEUF), using the gnomAD constraint metric, would amplify the contribution of variants in evolutionarily constrained genes and attenuate those in tolerant genes. A combined nonlinear composite of the form ∑2p(1−p) × [1 / (1 + exp(−γ(REVEL − θ)))] × (1 − LOEUF) would unite the population-genetics weighting validated here with the pathogenicity filter and the gene-context term, and is the immediate next formulation to be tested. Access to individual variant-level scores, rather than the gene-level aggregates used here, is a prerequisite for implementing the sigmoidal term correctly and would simultaneously resolve the aggregation limitation.

Beyond the formula itself, extending pathogenicity scoring to non-coding variants will require integrating complementary predictors where *REVEL* does not apply: CADD for regulatory and intronic variants, and SpliceAI for variants that disrupt pre-mRNA splicing. This is essential for the planned transition to whole-genome data through the CCDG Autism Whole Genome Sequence resource on the Terra/AnVIL platform, which will provide individual-level calls, non-missense variants, and the ability to stratify by clinical phenotype, such as the presence of intellectual disability or epilepsy. Finally, the metric is intended to support analysis at the level of gene sets and biological pathways rather than single genes, and to be applied across cohorts spanning evolutionary time. Stratifying genes by independent measures of selective pressure, including pLI, dN/dS ratios between humans and other primates, and overlap with human accelerated regions, would allow the central biological question motivating this work to be addressed: whether genes contributing to severe phenotypes are distinguished from those contributing to milder, polygenic presentations by their evolutionary trajectory. The preliminary three-cohort comparison incorporating ancient DNA, deferred here because of the ascertainment artifact in array-based ancient data, is the natural setting for that analysis once the nonlinear, context-aware metric and whole-genome data are in place.

We have presented and validated a composite gene-level pathogenicity metric that addresses a specific gap in existing variant-scoring approaches: the inability to compare pathogenic variant burden across collections of genes of different lengths, allele-frequency profiles, and variant counts, or between disease and population cohorts. The metric identifies elevated burden in the large majority of well-established autism genes, conditionally outperforms *REVEL*-based scoring alone, and provides the dynamic range needed for ranking. It is best understood not as a replacement for per-variant predictors but as a complementary gene-level layer that makes case–control and, ultimately, gene-set comparisons tractable and interpretable. Its current limitations, mainly the restriction to missense variants and the loss of information through linear aggregation, define a concrete roadmap toward a nonlinear, evolutionarily informed successor metric applied to whole-genome data, which is the subject of ongoing work.

## Data Availability

The 1000 Genomes Project Phase 3 data analyzed in this study are publicly available from the International Genome Sample Resource at https://www.internationalgenome.org/. The Simons Simplex Collection whole-genome sequencing data are subject to controlled access and cannot be redistributed by the authors; approved researchers may obtain the dataset (SFARI_SSC_WGS_1b) by application to SFARI Base at https://base.sfari.org, in accordance with the SFARI Researcher Distribution Agreement. REVEL scores are publicly available from the REVEL project website.

## Abbreviations

The following abbreviations are used in this manuscript:

LOEUF	Loss-of-function observed
gnomAD	Genome Aggregation Database
SFARI	Simons Foundation Autism Research Initiative
CADD	Combined Annotation Dependent Depletion
GSEA	Gene Set Enrichment Analysis
SKAT	Sequence Kernel Association Test
CMC	Combined Multivariate and Collapsing
CCDG	Centers for Common Disease Genomics
AF	Allele frequency

## Appendix A

Here we provide the final list of genes in our analysis as Appendix List A1.

Chromosome 1: CACNA1E; Chromosome 2: SCN2A, NRXN1, MBD5, SCN1A, MYT1L, NCOA1; Chromosome 3: FOXP1, CACNA1D, KAT2B, CACNA2D3; Chromosome 4: NR3C2, ANK2, FAM190A; Chromosome 5: TRIO, IQGAP2, GABRB2, RANBP17; Chromosome 6: SYNGAP1, ARID1B; Chromosome 7: CTTNBP2, KMT2C (MLL3), FOXP2, RELN; Chromosome 8: KCNQ3, DPYSL2; Chromosome 9: TEK, PAX5, PHF2, RFX3, RORB; Chromosome 10: TCF7L2, KCNMA1; Chromosome 11: KIRREL3, GALNTL4, SHANK2, LRRC4C; Chromosome 12: GRIN2B, MED13L, SLCO1B1, DNAH10; Chromosome 15: GABRB3, CHD2; Chromosome 16: ANKRD11; Chromosome 18: PTPRM, TCF4, CELF4, ASXL3; Chromosome 19: KDM4B, MYO9B; Chromosome 20: NINL; Chromosome 21: DIP2A, DYRK1A; Chromosome 22: TNRC6B.

As a clarifying note we wish to add that MLL3 is a legacy alias of KMT2C. The two identifiers entered the union independently because De Rubeis et al. reported the gene as MLL3 while Satterstrom et al. and Sanders et al. reported it as KMT2C. Both entries were retained as returned by the annotation pipeline and are identical in all computed values; their retention does not materially affect any reported statistic.

We also include the genes from each individual work as Appendix List A2.

De Rubeis et al. [22]: ADNP, ANK2, APH1A, ARID1B, ASH1L, ASXL3, ATP1B1, AXL, BCL11A, BIRC6, BRSK2, BRWD1, C11ORF30, CACNA1D, CACNA2D3, CAPN12, CARKD, CD163L1, CDC42BPB, CERS4, CHD8, CSNK1E, CSTF2T, CTTNBP2, CUL3, DNAH10, DPP3, DYRK1A, EP400, ETFB, FAMP190A, FCRL6, GABRB3, GALNTL4, GGNBP2, GRIN2B, IQGAP2, KATNAL2, KDM4B, KDM5B, KDM6B, KIAA0100, KIAA0182, KIRREL3, KLC1, KRT34, LEO1, MIB1, MLL3, MTMR12, MYH10, MYO9B, MYOC, MYT1L, NAA15, NCKAP1, NR3C2, NRXN1, PCOLCE, PHF15, POGZ, PPM1D, PRPF39, PTEN, PTPRM, RAB2A, RANBP17, RELN, S100G, SCARA3, SCN2A, SETD5, SHANK3, SIX2, SLC6A1, SLCO1B1, SLCO1B3, SMURF1, SPARCL1, STXBP5, SUV420H1, SYNGAP1, TAF4, TBR1, TCF3, TCTE3, TRIO, TTLL3, UBN2, UTP6, VIL1, WDFY3, WHSC1, ZNF774.

Satterstorm et al. [21]: ADNP, AID1B, ANK2, ANKRD11, AP2S1, ASH1L, ASXL3, BCL11A, CACNA1E, CACNA2D3, CELF4, CHD2, CHD8, CORO1A, CREBBP, CTNNB1, DEAF1, DIP2A, DNMT3A, DPYSL2, DSCAM, DYNC1H1, DYRK1A, EIF3G, ELAVL3, FOXP1, FOXP2, GABRB2, GABRB3, GFAP, GIGYF1, GNAI1, GRIA2, GRIN2B, HDLBP, HECTD4, IRF2BPL, KCNQ3, KDM5B, KDM6B, KIAA0232, KMT2C, KMT2E, KMT5B, KONMA1, LDB1, LRRC4C, MAP1A, MBD5, MED13L, MKX, MYT1L, NACC1, NCOA1, NR3C2, NRXN1, NSD1, NUP155, PAX5, PHF12, PHF2, PHF21A, POGZ, PPP2R5D, PPP5C, PRR12, PTEN, PTK7, RAI1, RFX3, RORB, SATB1, SCN1A, SCN2A, SETD5, SHANK2, SHANK3, SIN3A, SKI, SLC6A1, SMARCC2, SPAST, SRPRA, STXBP1, SYNGAP1, TAOK1, TBL1XR1, TBR1, TCF20, TCF4, TCF7L2, TEK, TLK2, TM9SF4, TRAF7, TRIM23, TRIP12, UBR1, VEZF1, WAC, ZMYND8.

Sanders et al. [20]: ACHE, ADNP, AKAP9, ANK2, APH1A, ARID1B, ASH1L, BLC11A, CAPN12, CHD2, CHD8, CTTNBP2, CUL3, DIP2A, DNMT3A, DSCAM, DYRK1A, ERBB2IP, ETFB, FOXP1, GABRB3, GIGYF1, GRIN2B, ILF2, INTS6, IRF2BPL, KAT2B, KATNAL2, KDM5B, KDM6B, KMT2C, KMT2E, MBD5, MFRP, MIB1, MYT1L, NAA15, NCKAP1, NINL, NLGN3, NRXN1, OR52M1, P2RX5, PHF2, POGZ, PTEN, PTK7, RANBP17, SCN2A, SETD5, SHANK2, SHANK3, SLC6A1, SPAST, SUV420H1, SYNGAP1, TBR1, TCF7L2, TNRC6B, TRIO, TRIP12, USP45, WAC, WDFY3, ZNF559.
